# Aplysin Retards Pancreatic Necrosis and Inflammatory Responses in NOD Mice by Stabilizing Intestinal Barriers and Regulating Gut Microbial Composition

**DOI:** 10.1155/2020/1280130

**Published:** 2020-08-01

**Authors:** Ying Liu, Xinyue Cui, Ming-Qing Gao, Meilan Xue, Hongwei Xu, Zhishang Chang, Yushan Jiang, Hui Liang

**Affiliations:** ^1^Basic Medical College, Qingdao University, Qingdao, 266071 Shandong, China; ^2^Department of Human Nutrition, College of Public Health, Qingdao University, Qingdao, 266071 Shandong, China; ^3^School of Medicine, Northwest University, Xi'an, 710069 Shaanxi, China; ^4^Laboratory of Biomedical Center, Qingdao University, Qingdao, 266021 Shandong, China

## Abstract

Aplysin is a brominated sesquiterpene with an isoprene skeleton and has biological activities. The purpose of this study is to investigate the inhibitory effect of aplysin on spontaneous pancreatic necrosis in nonobese diabetic (NOD) mice and its potential mechanisms. Results showed that NOD mice at 12 weeks of age showed obvious spontaneous pancreatic necrosis, damaged tight junctions of intestinal epithelia, and widened gaps in tight and adherens junctions. Aplysin intervention was able to alleviate spontaneous pancreatic necrosis in NOD mice, accompanied with decreased serum endotoxin levels and downregulated expressions of Toll-like receptor 4 and its related molecules MyD88, TRAF-6, NF-*κ*B p65, TRIF, TRAM, and IRF-3, as well as protein levels of interleukin-1*β* and interferon-*β* in pancreatic tissues. In addition, we observed obvious improvements of intestinal mucosal barrier function and changes of gut microbiota in the relative abundance at the phylum level and the genus level in aplysin-treated mice compared with control mice. Together, these data suggested that aplysin could retard spontaneous pancreatic necrosis and inflammatory responses in NOD mice through the stabilization of intestinal barriers and regulation of gut microbial composition.

## 1. Introduction

It has been reported that progressive pancreatic necrosis was involved in the development of pancreatitis and diabetes mellitus [1–3]. Therefore, it is important to inhibit pancreatic necrosis to prevent the progression of these diseases during the early stages of inflammatory response [4, 5]. Growing evidence has suggested that intestinal barriers and gut microbiota play pivotal roles in human health and disease [6, 7]. Changes in intestinal barrier integrity and gut microbial compositions contribute to a variety of metabolic and inflammatory diseases [8–10]. Endotoxin is a major component of cell walls of Gram-negative bacteria. Enhanced intestinal permeability, coupled with the overgrowth of Gram-negative bacteria in the gut, could lead to the production and leakage of large amounts of gut-derived endotoxins from the gut lumen into the systemic circulation [11]. These endotoxins result in the release of proinflammatory cytokines, such as interleukin- (IL-) 1*β* and interferon- (IFN-) *β*, which subsequently trigger or promote the development of local and systemic inflammatory responses via activating Toll-like receptor 4 (TLR4) signaling, including both TLR4-myeloid differentiation factor (MyD) 88 and TLR4-Toll-IL-1 receptor domain-containing adaptor inducing interferon-*β* (TRIF) pathways [12]. Multiple studies have suggested that gut microbial changes and intestinal barrier dysfunction were closely related to tissue necrosis in pancreas [6, 13]. Thus, modulation of the gut microbiota and maintenance of intestinal barrier integrity have emerged as potential therapeutic strategies for pancreatitis [14, 15].

**Figure 1:**
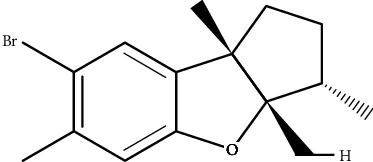
Chemical structure of aplysin.

Terpenoids from marine algae have excellent anti-inflammatory activity [16, 17]. Aplysin is a brominated sesquiterpene with an isoprene skeleton extracted from the red alga *Laurencia tristicha* (Figure 1). Aplysin is a naturally active marine substance with potential pharmacological activities, including hepatoprotective [18], immunoregulation [19], antitumor [20], and intestinal microregulatory properties [21]. However, it remains unclear whether aplysin has a potential to alleviate pancreatic necrosis in the process of pancreatitis.

In this study, we investigated the protective efficacy of aplysin against the spontaneous pancreatic necrosis and inflammatory responses by stabilizing intestinal barriers and regulating gut microbial composition in nonobese diabetic (NOD) mice.

## 2. Materials and Methods

### 2.1. Extraction, Purification, and Identification of Aplysin

As described in our previous publications [22–24], aplysin was extracted and purified from red alga *L*. *tristicha* collected on the Naozhou Island coast of Zhanjiang City, China, and it was identified by Dr. Ding Lanping from Institute of Oceanology, Chinese Academy of Sciences. In brief, the air-dried red alga *L*. *tristicha* was extracted using ethyl alcohol at room temperature, and the extract was concentrated under reduced pressure at a temperature below 40°C. Then, the residue was partitioned with ethyl acetate, chromatographed over silica gel, and eluted with a gradient increase of ethyl acetate from 0 to 100% in light petroleum. Thin-layer chromatography analysis was used to detect the components. These components were eluted by pure light petroleum followed by recrystallization to give a colorless needle crystal. Finally, the compounds were analyzed by infrared radiation (IR), electron impact-mass spectrometry (EI-MS), ^1^H-nuclear magnetic resonance (NMR), and ^13^C-NMR.

### 2.2. Animal Experiments

Female NOD mice aged 6 weeks were purchased from Beijing Vital River Laboratory Animal Technology Co., Ltd. (License No.: SCXK [Jing], 2016-0006). Mice were maintained in an animal facility under a set temperature of 22°C–25°C and a relative humidity of 50%–60% with a 12 h light-dark cycle. Mice were housed in the specific pathogen-free animal center of Qingdao University with ad libitum access to standard irradiated rodent chow and sterile tap water. After a 2-week acclimatization period, NOD mice were intragastric gavaged with 150 mg·kg^−1^ aplysin dissolved in soya bean oil once daily (*n* = 15). Mice intragastric gavaged with an equal volume of soya bean oil were used as controls (*n* = 15). After 4 weeks of treatment, mice were anesthetized by intraperitoneal injection of 40 mg·kg^−1^ sodium pentobarbital. The blood, pancreas, jejunum, colon, and colonic contents were collected for further analysis. All protocols were approved by the Animal Ethics Committee of Qingdao University. Experimental animals were cared for according to the International Guide for Care and Use of Laboratory Animals.

### 2.3. Histopathology

Pancreas samples were fixed with 4% paraformaldehyde for >24 h, embedded in paraffin, and cut into 5 *μ*m thick sections using an RM 2135 rotary microtome (Leica, Wetzlar, Germany). Sections were stained with hematoxylin and eosin using a standard procedure. Slides were observed and photographed using a light microscope (Olympus BX50, Tokyo, Japan).

### 2.4. Transmission Electron Microscopy

Pancreatic, jejunal, and colonic tissues were cut into 1 mm × 2 mm blocks and fixed with 2.5% glutaraldehyde at 4°C for >24 h. All tissue samples were washed three times with phosphate-buffed saline (PBS), postfixed with 1% osmium tetroxide for 80 min, dehydrated in a standard series of acetone concentrations, and then embedded into epoxy resin (SPI Chem/SPI-PON 812 KIT, West Chester, PA). Subsequently, semithin sections (1 *μ*m) of embedded tissue were treated with 1% toluidine blue to assess localization; then, ultrathin sections (70 nm) were created and collected on 150-mesh grids and stained with 3% uranyl acetate and lead citrate. The ultrastructures of the jejunal and colonic epithelial cell connections were examined and photographed using a JEM-1200EX transmission electron microscope (JEOL, Tokyo, Japan).

### 2.5. ELISA

Serum endotoxin and insulin levels were measured with immunoassay kits (USCN KIT INC., Wuhan, Hubei, China) in accordance with the manufacturer's instructions.

### 2.6. Immunofluorescence

Pancreas, jejunum, and colon tissue samples were fixed in 4% paraformaldehyde overnight, embedded in paraffin, and cut into 5 *μ*m thick sections. The sections were blocked with 5% normal goat serum in PBS for 30 min at 4°C, then incubated with specific primary antibodies overnight at 4°C. The primary antibodies were polyclonal goat anti-insulin, polyclonal goat anti-occludin, polyclonal goat anti-zonula occludens- (ZO-) 1, or polyclonal mouse anti-claudin-1 (Cell Signaling Technology, Danvers, MA). After washing three times with PBS, the sections were incubated with a corresponding secondary antibody (Zymed Laboratories, Santiago, CA) at 37°C in the dark for 60 min. The nucleus was stained using 4′,6-diamidino-2-phenylindole. The distributions of insulin, occludin, ZO-1, and claudin-1 proteins in pancreatic tissue and intestinal epithelial cells were observed and photographed using a TCS SP8 CARS Laser Scanning confocal microscope (Leica, Wetzlar, Germany).

### 2.7. Western Blotting

Total protein was extracted from pancreatic, jejunal, and colonic tissues using membrane and cytoplasmic or nuclear and cytoplasmic protein extraction kits (Beyotime, Jiangsu, China) following the manufacturer's instructions. Equal amounts of protein for each sample were subjected to 5%–10% SDS-PAGE and then transferred onto polyvinylidene difluoride membranes (Millipore, Bedford, MA). The membranes were blocked with 10% nonfat milk in Tris-buffered saline containing 0.1% (*v*/*v*) Tween-20 and incubated with primary antibodies against TLR4, MyD88, TNF receptor associated factor- (TRAF-) 6, IL-1*β*, TRIF, TRIF-related adaptor molecule (TRAM), IFN-*β* (Bioss, Beijing, China), nuclear factor- (NF-) *κ*B p65 (Cell Signaling Technology), interferon regulatory factor- (IRF-) 3 (Santa, Dallas, TX), claudin-1, occludin, ZO-1 (Sangon, Shanghai, China), *β*-actin, histone H3 (Cell Signaling Technology), or Na/K ATPase (Proteintech, Rosemont, IL) at 4°C overnight. Subsequently, membranes were washed before incubation with the corresponding secondary antibodies (Zhongshan Goldenbridge, Beijing, China) for 2 h at 37°C. Finally, protein bands were visualized using an enhanced chemiluminescence detection kit (Beyotime). Na/K ATPase, *β*-actin, and histone H3 served as internal controls for membrane proteins, cytoplasmic proteins, and nucleoproteins, respectively.

### 2.8. Tracer Experiment

Sections of the jejunum and colon were obtained from the middle and ligated at one end. Then, EZ-Link Sulfo-NHS-Biotin (Pierce Chemical, Rockford, IL) was slowly injected into the intestine samples from the other end, which was then ligated. The samples were incubated for 5 min at room temperature, then fixed in 4% paraformaldehyde for >3 h. After washing three times with ice-cold PBS, 5 *μ*m thick frozen tissue sections were prepared and incubated with streptavidin conjugated to Alexa Fluor 488 for 30 min. Finally, the distribution of biotin in the intestine was observed and photographed with a BX53 fluorescence microscope (Olympus, Tokyo, Japan).

### 2.9. DNA Extraction and 16S rRNA Sequencing

16S rRNA gene sequencing was performed by Realbio Genomics Institute (Shanghai, China). Genomic DNA was extracted from the colon contents of the NOD mice with a QIAamp Fast DNA Stool Mini Kit (QIAGEN, Hilden, Germany) following the manufacturer's protocols. Amplification of the V3-V4 regions of the 16S ribosomal RNA gene, quantitation, sequencing, and analysis were performed following our previously published method and using the Illumina HiSeq PE250 sequencing platform (Illumina, Inc., San Diego, CA, USA) [25]. We have registered the raw sequence reads of the gut microbial contents in the Sequence Read Archive database of GenBank, under accession number PRJNA598993. We used Chao1 and Shannon diversity indices for comparison of bacterial operational taxonomic unit (OTU) richness and evenness of the gut microbiota, respectively. Principal coordinate analysis (PCoA) was performed on weighted UniFrac distances of the relative OTU abundance to visualize the segregation of gut microbiota structures between the two groups. Analyses of similarity (ANOSIM) were performed to calculate *R* and *p* values using phylogeny-based weighted UniFrac distance metrics to test the similarity of the gut microbiota between groups.

### 2.10. Statistical Analysis

Statistical analysis was performed using SPSS 17.0 and GraphPad Prism 5. Quantitative data were expressed as means ± standard deviations. Differences between parametric variables were assessed using Fisher's least significant difference test. Differences between nonparametric variables were analyzed using the Kruskal–Wallis and Mann–Whitney tests. Values of *p* < 0.05 were considered statistically significant.

## 3. Results

### 3.1. Extraction and Identification of Aplysin

The molecular formula of the extract from red alga *L*. *tristicha* in this study is C_15_H_19_OBr, and its the molecular weight is 295. It was identified as aplysin based on the spectral and physical characteristics by comparison with the corresponding literature data [26, 27] (Supplementary data 1).

### 3.2. Aplysin Treatment Alleviated Spontaneous Pancreatic Necrosis in NOD Mice

**Figure 2:**
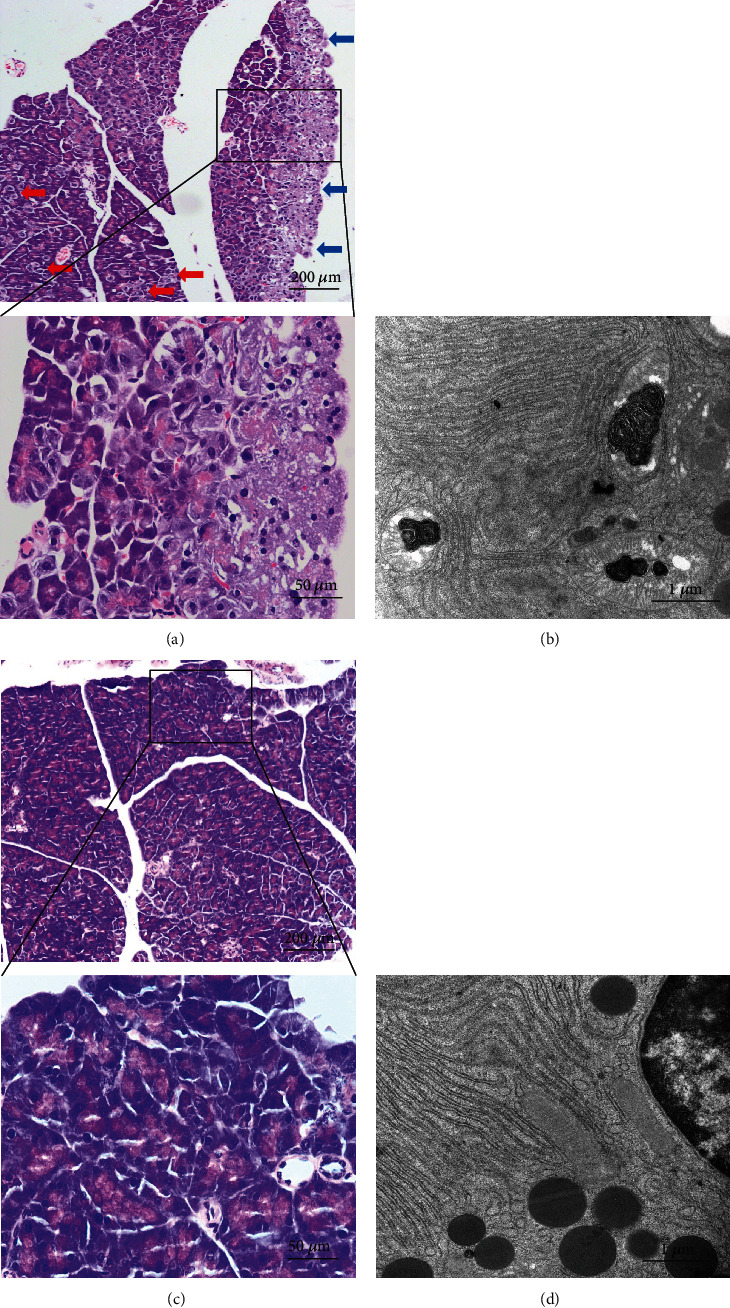
Representative images of pancreatic tissue sections after hematoxylin and eosin staining. (a, c) Tissue sections were examined under a phase-contrast microscopy after hematoxylin and eosin (H&E) staining. (b, d) Tissue sections were examined under a transmission electron microscope. The red arrows indicate focal necrosis, and the blue arrows indicate large patchy necrosis.

At 12 weeks of age, NOD mice in the control group showed spontaneous diffuse necrosis of pancreatic acinar cells, as indicated by swelling, hydropic degeneration, membrane rupture, and vacuolation of pancreatic acinar cells in the tissue sections of exocrine pancreas examined by hematoxylin and eosin. Mat striatal fibrosis was also observed in the edge of the pancreas (Figure 2(a)). In addition, dilation and disorderly arrangement of endoplasmic reticulum, swelling and vacuolization formation of mitochondria, and depletion in numbers of lysosome were observed in NOD mice in the control group (Figure 2(b)). However, necrotizing lesions of pancreatic acinar cells were alleviated in NOD mice treated with aplysin compared with control mice (Figures 2(c) and 2(d)).

### 3.3. Aplysin Treatment Decreased Serum Endotoxin Levels

**Figure 3:**
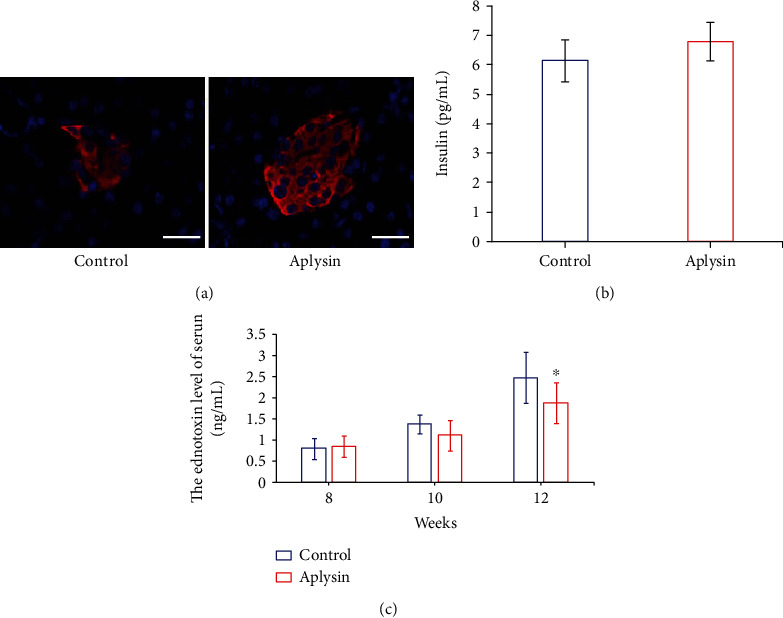
Levels of insulin in the pancreas and the serum and endotoxin in the serum. (a) Immunofluorescence staining of insulin in the pancreas. Scale bar = 40 *μ*m. Red: insulin distribution; blue: cell nuclei stained with DAPI. (b) Insulin levels in serum of mice assessed by ELISA. (c) Endotoxin in serum of mice assessed by ELISA. ^∗^*p* < 0.05 vs. the control.

Aplysin treatment did not change insulin expression by islet cells in tissue sections (Figure 3(a)) and insulin levels in serum of mice (Figure 3(b)) compared with that in mice in the control group at 12 weeks of age.

In addition, no difference was observed at the initial levels of endotoxin between these two groups at 8 weeks of age. However, aplysin treatment obviously reduced the endotoxin levels in serum of mice compared with that in the control group at 12 weeks of age (*p* < 0.05, Figure 3(c)).

### 3.4. Aplysin Treatment Downregulated Expressions of TLR4 and Its Downstream Effectors in Pancreatic Tissues

**Figure 4:**
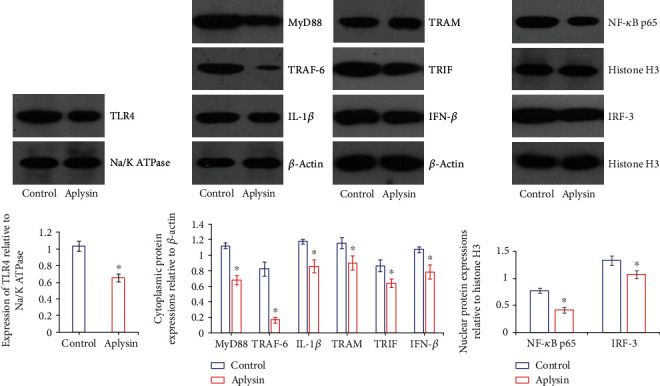
Expressions of TLR4 and its downstream effectors in pancreatic tissues. Representative blots for TLR4, MyD88, TRAF-6, IL-1*β*, TRAM, TRIF, IFN-*β*, NF-*κ*B p65, and IRF-3 in the pancreas of aplysin-treated or untreated mice were analyzed by Western blotting. Na/K ATPase, *β*-actin, and histone H3 were used as internal controls for membrane proteins, cytoplasmic proteins, and nucleoproteins, respectively. ^∗^*p* < 0.05 vs. the control group.

Results of Western blotting showed that aplysin treatment obviously downregulated the expression of TLR4, as well as its downstream effectors MyD88 and TRIF, in the pancreatic tissues of NOD mice. Expressions of MyD88-associated molecules TRAF-6 and NF-*κ*B p65, and TRIF-associated molecules TRAM and IRF-3, were also downregulated in pancreatic tissues of mice following aplysin treatment. Furthermore, aplysin treatment resulted in obvious downregulations of IL-1*β* and IFN-*β* protein expressions in pancreatic tissues (Figure 4).

### 3.5. Aplysin Treatment Enhanced Intestinal Barrier Integrity

**Figure 5:**
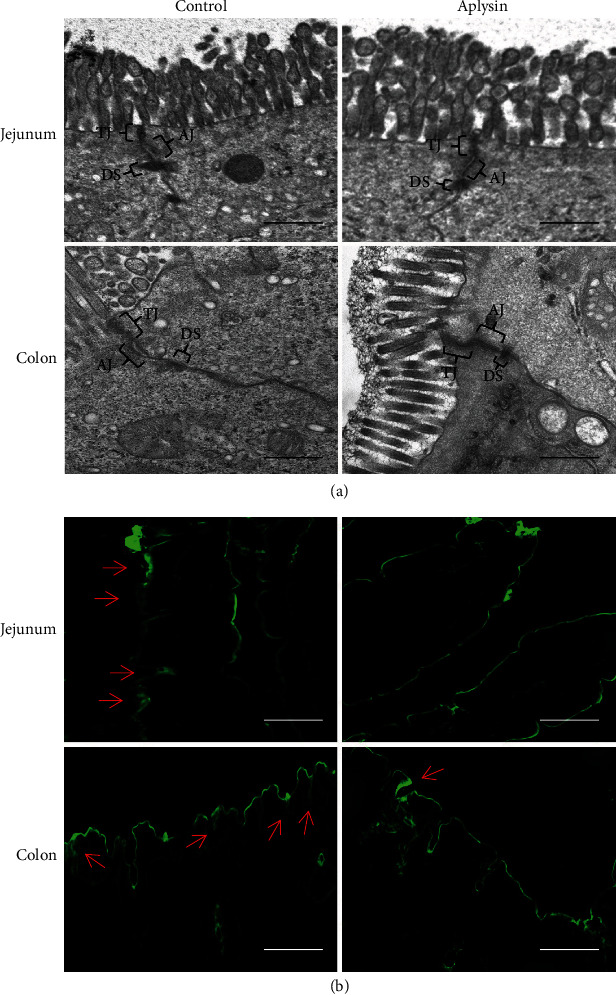
The permeability analysis of the jejunum and colon of mice. (a) The ultrastructure of the jejunum and colon examined by transmission electron microscopy. TJ: tight junction; AJ: adherens junction; DS: desmosomes. Scale bar = 400 nm. (b) The permeability assessment of the jejunum and colon by tracer experiments. The green fluorescence signals indicate biotin distribution; the red arrows indicate tracer leakage. Scale bar = 50 *μ*m.

**Figure 6:**
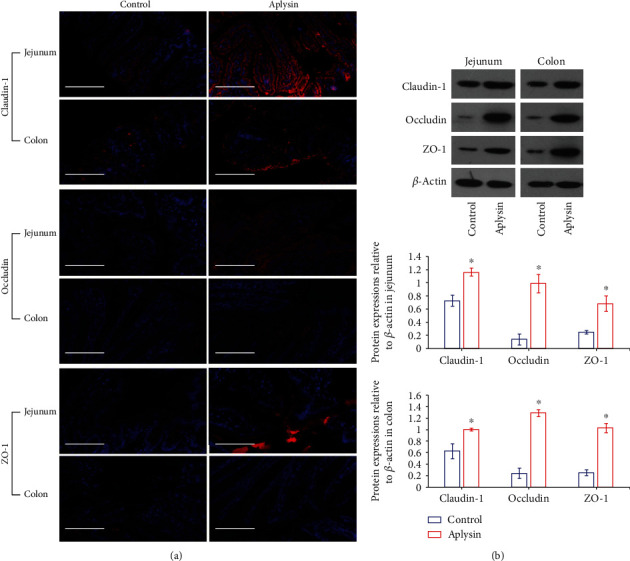
Expressions of tight junction-related proteins in tissues of the jejunum and colon. (a) Expressions of claudin-1, occludin, and ZO-1 protein examined by immunofluorescence assay. Nuclei were stained with DAPI. Scale bar = 50 *μ*m. (b) Expressions of claudin-1, occludin, and ZO-1 protein examined by Western blot. *β*-Actin was used as a loading control. ^∗^*p* < 0.05 vs. the control.

The ultrastructures of jejunal and colonic epithelial cells under transmission electron microscopy showed that the tight junctions were damaged in the control group, and the gaps of tight and adherens junctions were abnormally widened. Following aplysin treatment for 4 weeks, the intercellular junction structure of epithelial cells was basically normal (Figure 5(a)). Meanwhile, biotin tracer experiments showed that biotin fluorescent signals were restricted to the jejunal and colonic lumen in the aplysin group, while there was a different degree of infiltration of biotin fluorescent signals in the jejunum and colon of the control group (Figure 5(b)). Furthermore, immunofluorescence staining showed that the tight junction-related proteins claudin-1, occludin, and ZO-1 were present at the surface of columnar epithelial cells in the jejunum and colon of both groups; however, their expression levels were higher in the aplysin treatment group than those in the control group (Figure 6(a)). Western blot analysis also showed that expression of these molecules was obviously upregulated in the aplysin group compared with the control group (Figure 6(b)).

### 3.6. Gut Microbiota Analysis of NOD Mice by the Illumina HiSeq Sequencing Platform

**Figure 7:**
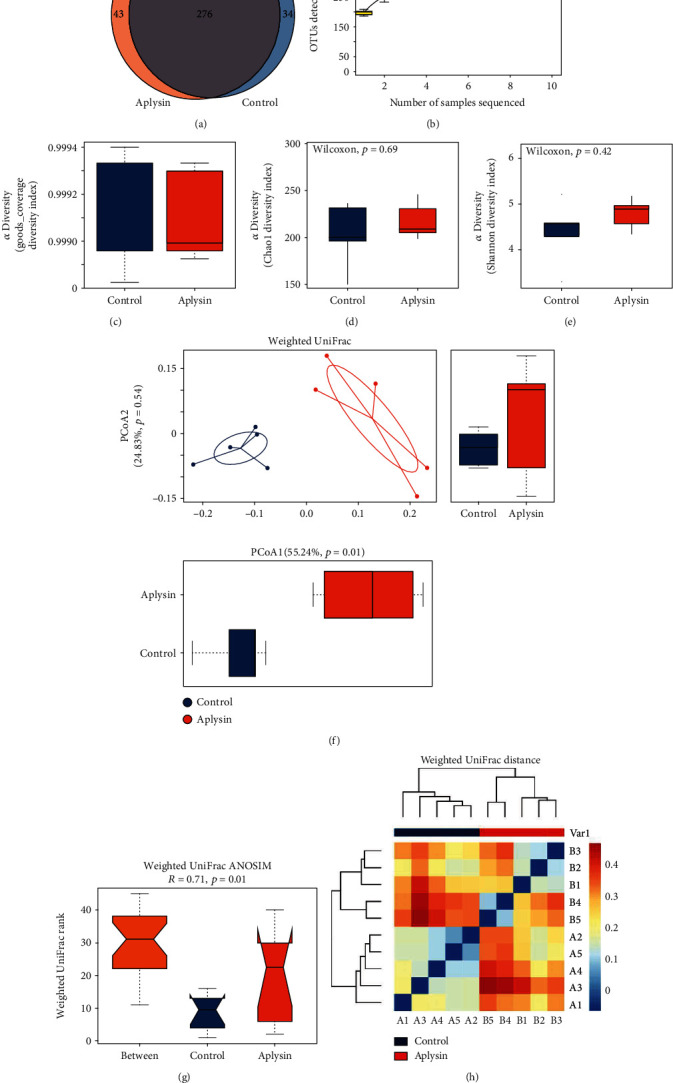
The diversity analysis of the intestinal flora of mice. (a) A Venn diagram showing the number of the common and unique bacterial OTUs between the control and aplysin intervention groups. (b) Species accumulation curves and (c) goods_coverage assessing sequencing depth. (d) The Chao1 showing species richness of the intestinal flora. *p* > 0.05 indicates that there was no obvious difference between groups. (e) The Shannon index showing species evenness. *p* > 0.05 indicates that there was no obvious difference between groups. (f) Principal coordinate analysis (PCoA) of intestinal flora among all samples. Cluster of samples implies their species compositions were similar or identical. *p* < 0.05 indicated that the difference between the two groups for the corresponding principal coordinate was obvious. (g) ANOSIM (analysis of similarity). *R* > 0 and *p* < 0.05 indicated an obvious difference between these two groups. (h) The heat map based on weighted UniFrac distances of the 10 samples. Proximity of samples indicated similar species compositions.

Using Illumina HiSeq sequencing of the bacterial 16S rRNA gene V3-V4 regions, 571,933 clean reads were obtained from 10 colon content samples, resulting in an average yield of 57,193 ± 6229 clean tagged reads per sample. After random subsampling, 33,878 reads were obtained from each sample, and 1939 OTUs were obtained from all samples. A Venn diagram showed that 276 OTUs were common to both groups, 34 OTUs were unique to the control group, and 43 OTUs were unique to the aplysin treatment group (Figure 7(a)). At this sequencing depth, species accumulation curves plateaued, and the goods_coverage index was >99% in both groups indicating that most phylotypes had already been covered (Figures 7(b) and 7(c)).

### 3.7. Comparison of the Microbial Community Structures in Mice with and without Aplysin Treatment

Analysis of the Chao1 index and the Shannon index suggested that the gut microbiota richness and evenness in the aplysin treatment group was slightly increased compared with the control group, although this difference did not reach statistical significance (*p* > 0.05, Figures 7(d) and 7(e)).

In addition, we found that based on PCoA1 (55.24% of variance explained, *p* = 0.01), the weighted UniFrac PCoA scores clearly separated samples from aplysin-treated mice from those of control mice (Figure 7(f)). Subsequent weighted UniFrac ANOSIM analysis confirmed a statistically obvious separation between the gut microbial communities of the control mice and the aplysin-treated mice (*R* = 0.71, *p* = 0.01, Figure 7(g)). Furthermore, a heat map based on weighted UniFrac distances showed that NOD mice treated with aplysin had dissimilar microbial compositions compared with untreated NOD mice (Figure 7(h)).

**Figure 8:**
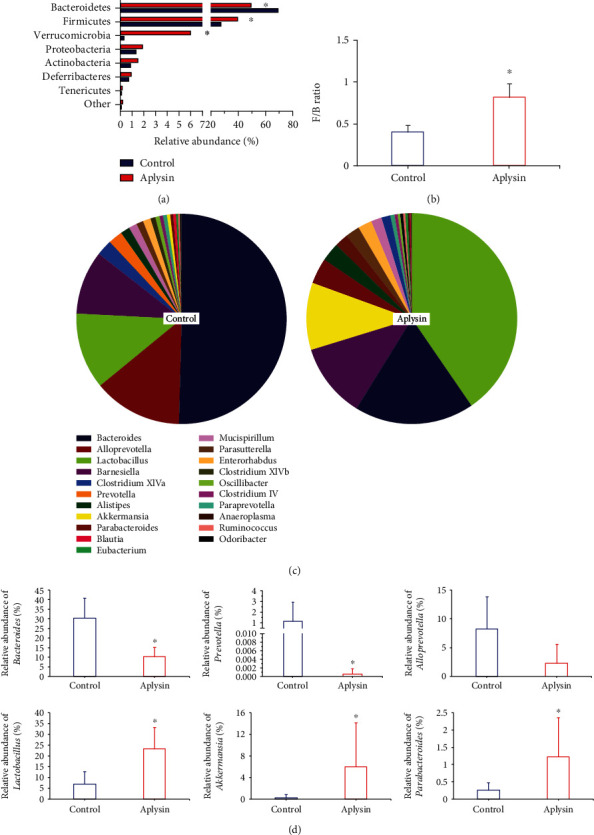
Analysis of the structure and composition of intestinal flora of mice. (a) Analysis of the relative abundance of bacteria at the phylum level. ^∗^*p* < 0.05 vs. the control. (b) Analysis of the *Firmicutes*/*Bacteroidetes* (F/B) ratio of intestinal flora. ^∗^*p* < 0.05 vs. the control. (c) Composition analysis of the dominant genera (top 20). (d) Changes in relative abundance of several important genera, including *Bacteroides* (upper left), *Lactobacillus* (bottom left), *Akkermansia* (bottom middle), *Prevotella* (upper middle), *Parabacteroides* (bottom right), and *Alloprevotella* (upper right), in intestinal flora. ^∗^*p* < 0.05 vs. the control.

At the phylum level, *Bacteroidetes* and *Firmicutes* were the most abundant phyla in both groups. The relative abundance of *Bacteroidetes* was lower in mice treated with aplysin (49.54%) than in the control group (69.17%), while the relative abundance of *Firmicutes* was increased (39.77%) in aplysin-treated mice compared with the control group (27.41%) (Figure 8(a)). We further performed ratio analyses of *Firmicutes*/*Bacteroidetes* (F/B) comparing both groups. As seen in Figure 8(b), the F/B ratio was markedly increased in the aplysin treatment group compared with the control group (0.82 and 0.40, respectively, *p* < 0.05). In addition, the relative abundance of *Verrucomicrobia* was obviously increased in aplysin-treated mice (6.00%) compared with untreated mice (0.30%) (*p* < 0.05).

At the genus level, analysis of the dominant genera (top 20) indicated that the most abundant genus was *Bacteroides* in control mice, while the most abundant genus was *Lactobacillus* in aplysin-treated mice (Figure 8(c)). The relative abundance of both *Bacteroides* (control vs. aplysin: 30.59% vs. 10.40%) and *Lactobacillus* (control vs. aplysin: 7.14% vs. 23.28%) was obviously different between the two groups (*p* < 0.05) (Figure 8(d)). Meanwhile, *Akkermansia* and *Parabacteroides* were enriched in aplysin-treated mice compared with control mice (Figure 8(c)). The abundances of *Akkermansia* and *Parabacteroides* were 6.00% and 1.23%, respectively, in the aplysin-treated group, and were both obviously higher than those in the untreated group (*Akkermansia*: 0.30%, *Parabacteroides*: 0.27%) (*p* < 0.05, Figure 8(d)). *Prevotella* was the sixth most abundant genus (top 20) in the control group (Figure 8(c)), with a relative abundance of 1.32% in the gut microbiota of untreated NOD mice (Figure 8(d)). However, *Prevotella* were almost undetectable in aplysin-treated mice (*p* < 0.05, Figures 8(c) and 8(d)).

**Figure 9:**
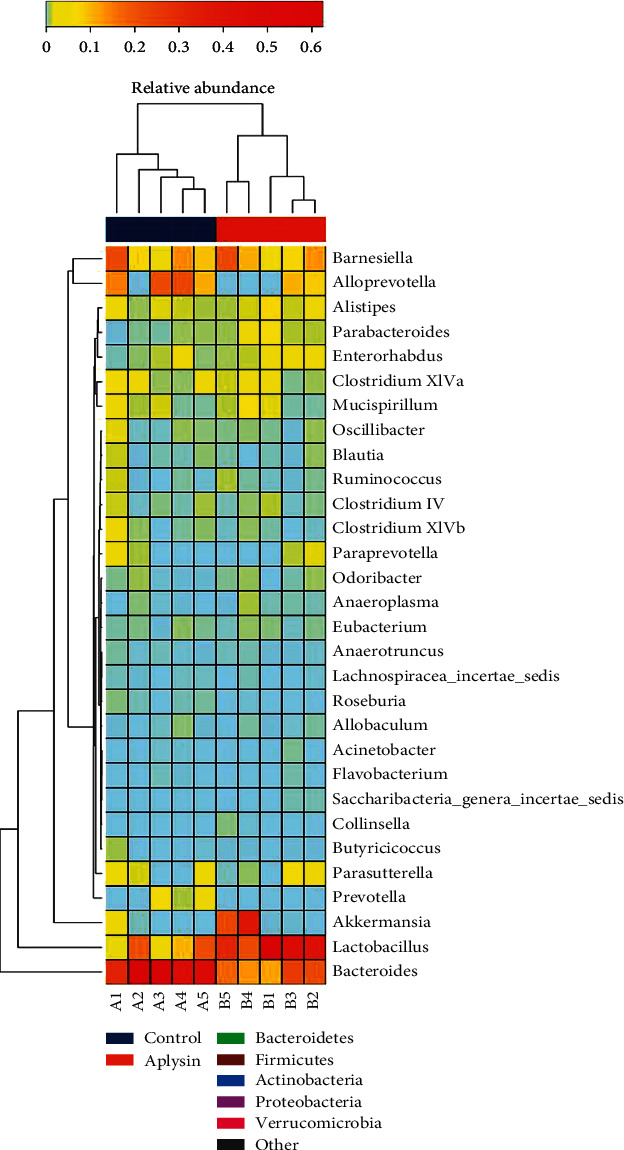
Heat map comparison at the genus level (top enriched 30 genus) of intestinal flora in each sample. The phylogenetic relationships, clustering analysis, and the phylum to which each genus belonged in both groups are displayed.

Heat map analysis at the genus level (top 30) showed that the effect of aplysin on *Firmicutes* mainly involved the dominant genera such as *Lactobacillus* but that aplysin treatment led to obvious changes in the abundances of multiple *Bacteroidetes* genera including *Bacteroides*, *Prevotella*, *Parabacteroides*, and *Alloprevotella* (Figure 9).

## 4. Discussion

NOD mice have been widely used as a model to study T1DM. These mice can spontaneously develop T1DM, with symptoms similar to those of T1DM in humans [28]. An intensifying inflammatory response is accompanied by progressive pancreatic necrosis, which greatly promotes the pathological progression of NOD mice to T1DM [29]. We used NOD mice as an experimental model to investigate the inhibitory effects of aplysin on pancreatic necrosis in this study. Pancreatic acinar cell necrosis is considered an initial event in pancreatitis; histopathologically, the cytoplasmic vacuolization of acinar cells is the main manifestation and typical feature. We demonstrated that aplysin intervention obviously alleviated pancreatic acinar cell necrosis in NOD mice, which suggested aplysin might be used to treat pancreatitis as a drug.

The translocation of gut-derived endotoxins following intestinal barrier dysfunction and intestinal Gram-negative bacteria overgrowth is a key event contributing to pancreatitis [12, 30, 31]. The elevated level of serum endotoxin is an important marker of intestinal barrier dysfunction, and circulating endotoxin is involved in the development of systemic inflammatory response. Schietroma et al. demonstrated a clear increase of serum endotoxin in 63 patients with pancreatitis compared with the control group; furthermore, the patients with severe pancreatitis had higher serum endotoxin as regards mild pancreatitis [30]. Shen et al. found that the serum inflammatory factor levels reduced obviously with the decrease of serum endotoxin after intervention by early enteral nutrition in pancreatitis patients [31]. Hence, we further detected serum endotoxin, intestinal barrier function, and gut microbiota to investigate the potential mechanisms in which aplysin retards the spontaneously pancreatic necrosis in NOD mice. We found that levels of serum endotoxin were obviously decreased in NOD mice treated with aplysin compared with untreated mice, which indicated that aplysin can ameliorate pancreatic necrosis by lessening circulating endotoxin levels.

TLR4 is an essential endotoxin signaling receptor, and the activation of TLR4 signaling (TLR4/MyD88 and TLR4/TRIF) plays a crucial role in the development of inflammatory responses [12]. In the present study, we found that the decreased level of serum endotoxin was accompanied by the downregulated expressions of TLR4 and its downstream effectors MyD88, TRIF, TRAF-6, NF-*κ*B p65, TRAM, and IRF-3 and reduced release of proinflammatory mediators IL-1*β* and IFN-*β* in the pancreas of NOD mice. These findings were consistent with other relevant reports [32, 33].

Several studies in humans have shown that patients with pancreatitis have intestinal barrier dysfunction [30] accompanied by lower levels of occludin and ZO-1 [34], both of which contribute to the stability of tight connections between cells as tight connection-related proteins. Similar conclusions have been reached using animal models [35]. Early intervention contributed to improving intestinal barrier function and promoting recovery from inflammation in patients and animals with pancreatitis [31, 36]. In the present study, we analyzed barrier function of the jejunum and colon epithelial cells using transmission electron microscopy, tracer experiments, immunofluorescence, and Western blotting to observe changes in intestinal mucosal epithelial cell junctions (such as tight junctions, adherens junctions, and desmosomes), intestinal permeability, and expression of tight connection-related proteins. Intestinal barrier integrity was damaged in untreated NOD mice, but after intervention with aplysin, the impaired intestinal barrier was effectively repaired, with increased expression of claudin-1, occludin, and ZO-1 observed in the jejunum and colon compared with untreated mice. These results, together with decreased levels of endotoxin, indicated that aplysin decreased endotoxin leakage by improving intestinal barrier function; this might represent one mechanism through which aplysin alleviated pancreatic necrosis.

The role of the gut microbiota in the development of pancreatitis is increasingly emphasized. Both human and experimental studies have demonstrated and confirmed that pancreatitis is often closely associated with gut microbiota disturbances [37, 38]. In the current study, we found that mice treated with aplysin had distinct gut microbial profiles compared with untreated mice, although differences in richness and evenness in the gut microbiota were not obvious between the two groups.

At the phylum level, the relative abundance of *Verrucomicrobia* was higher in aplysin-treated mice compared with untreated mice. Even more remarkably, the relative abundance of *Bacteroidetes* was obviously decreased and the proportion of *Firmicutes* was obviously elevated in aplysin-treated NOD mice, and the *Firmicutes*/*Bacteroidetes* ratio was increased. *Firmicutes* and *Bacteroidetes* were the two most abundant phyla in gut microbiota; the *Firmicutes* are mostly Gram-positive bacteria, and the *Bacteroidetes* are mostly Gram-negative bacteria. Thus, the *Firmicutes*/*Bacteroidetes* ratio partially reflects the status of gut microbiota [39]. Our studies indicated that changes in the composition of gut microbiota occurred in aplysin-treated mice compared with untreated mice.

At the genus level, there was an obviously reduced relative abundance of *Bacteroides* (*Bacteroidetes*) in aplysin-treated NOD mice. The *Bacteroides* are Gram-negative, obligatory anaerobic bacilli, and the proportion of *Bacteroides* among intestinal anaerobes is about 25%. *Bacteroides* species are normally mutualistic in the gut microbiota [40]. However, these bacteria can change from friendly symbionts to dangerous pathogens by turning on expression of certain genes [41]. For example, *Bacteroides fragilis*, the most virulent member of the *Bacteroides*, can contribute to infections with high morbidity and mortality [42]. Furthermore, tissue destruction can be mediated by histolytic enzymes produced by *B*. *fragilis*, and the tight junctions of the intestinal epithelium can be destroyed by the enterotoxin and the endotoxin produced by this organism. This results in rearrangements of the epithelial cell cytoskeleton and loss of tight junctions to cause intestinal leakage [43, 44]. Previous studies have shown that an increased relative abundance of *Bacteroides* was observed in mice with chronic pancreatitis [8]. In addition, pancreatic sepsis caused by *Bacteroides* is one of the most lethal complications of acute pancreatitis [45]. Similar conclusions were reached in our study. Moreover, we found that the relative abundance of *Prevotella* (*Bacteroidetes*) was obviously reduced after treatment with aplysin. Multiple studies have shown that the *Prevotella* were responsible for secondary infections in certain inflammatory diseases such as periodontitis, rheumatoid arthritis, and acute necrotizing pancreatitis, which can be treated with antibiotics [46]. Our results showed that aplysin protects against spontaneous pancreatic necrosis in NOD mice related to the reduced relative abundance of some certain Gram-negative pathogens such as *Bacteroidetes* and *Prevotella*. In addition, our results showed that *Lactobacillus* (*Firmicutes*) and *Akkermansia* (*Verrucomicrobia*) were obviously enriched in aplysin-treated NOD mice (>3.5-fold and nearly 20-fold more abundant in the gut microbiota of aplysin-treated mice compared with untreated mice, respectively). It should be noted that these species have been regarded as beneficial microbes for health [47, 48]. *Lactobacillus* can regulate the structure of the gut microbiota, limit the intestinal adhesion and overgrowth of potential pathogens, and improve intestinal barrier function [49, 50]. *Akkermansia* is specialized in mucus utilization [51] and also has potential roles in repairing impaired intestinal barriers [52], reducing endotoxemia to regulate inflammatory disorders by increasing mucus layer thickness and antagonizing gut pathobiont colonization [53, 54]. Notably, Hänninen and colleagues [53] showed that *Akkermansia muciniphila* could delay T1DM development in NOD mice. Our results suggested that the probiotics such as *Akkermansia* and *Lactobacillus* involved in the protective effect of aplysin on spontaneous pancreatic necrosis in NOD mice.

In summary, our findings provide evidence that pancreatic necrosis was obviously delayed in NOD mice treated with aplysin through stabilization of intestinal barriers and alteration of gut microbial composition. To our knowledge, this is the first study showing that aplysin treatment effectively protects against spontaneous pancreatic necrosis and inflammatory responses in NOD mice. These novel findings would provide a foundation for the reasonable supplement of marine terpenoids to treat pancreatitis.

## Data Availability

The data of raw sequence of the gut microbial contents used to support the findings of this study have been deposited in the GenBank database under accession number PRJNA598993.

## Supplementary Materials

Supplementary Materials Supplementary data 1: components of aplysin structure analyzed by infrared radiation (IR), electron impact-mass spectrometry (EI-MS), ^1^H-nuclear magnetic resonance (NMR), and ^13^C-NMR.
