# Supplementary material for: Aplysin Retards Pancreatic Necrosis and Inflammatory Responses in NOD Mice by Stabilizing Intestinal Barriers and Regulating Gut Microbial Composition
Source: Mediators Inflamm. 2020 Aug 1;2020:1280130. doi: 10.1155/2020/1280130 (PMC7416259; doi:10.1155/2020/1280130)
Supplement: Supplementary Materials — Supplementary data 1: components of aplysin structure analyzed by infrared radiation (IR), electron impact-mass spectrometry (EI-MS), 1H-nuclear magnetic resonance (NMR), and 13C-NMR. [file 1280130.f1.pdf]

Supplementary data 1:

Components of aplysin structure analyzed by infrared radiation (IR), electron impact-mass spectrometry (EI-MS), 1H-nuclear magnetic resonance (NMR) and 13C-NMR.

IR ν_{max}^{KBr} cm^{-1}: 2 952, 2 864, 1 577, 1 487, 1 460, 1 375, 1 308, 1 267, 1 234, 1 192, 1 007, 904, 881, 862.

EI-MS *m /z* (%): 296 [M (^{81} Br)]$^{+}$ (100), 294 [M(^{79} Br)]$^{+}$ (100) , 281 [M(^{81} Br) - CH_3]$^{+}$ (95), 279 [M(^{79} Br) - CH_3]$^{+}$ (95) , 239(45) , 237(45) , 200 [M-Br]$^{+}$(35) , 160 (16) , 115 (12) , 109 (10) , 69 (5).

^{1} H-NMR(CD_3 $COCD_3$, 500 MHz)W: 1. 06(3H, d, *J*= 6. 5 Hz, H-9) , 1. 04～ 1.08 (1H, m, H-2a) , 1. 27(3H, s, H-10) , 1. 33(3H, s, H-12) , 1. 60～1. 68(2H, m, H-2b, 1a) , 1. 81～1. 86(2H, m, H-1b, 3) , 2. 26(3H, s, H-11) , 6. 60(1H, s, H-5) , 7. 2(1H, s, H-8).

^{13}C-NMR(CD_3 $COCD_3$, 125 MHz)W: 43. 0(C-1) , 31. 9 (C-2) , 46. 6 (C-3) , 100. 4 (C-3a) , 159. 4 (C-4a) , 110. 5(C-5) , 137. 5(C-6) , 114. 3(C-7) , 127. 3(C-8) , 137. 4(C-8a) , 55. 1(C-8b) , 13. 3(C-9) , 23. 1(C-10) , 23. 4(C-11) , 20. 1(C-12).

By comparing with corresponding literature data [1,2], this compound was identified as aplysin.

References

[1] J. Y. Laronze, R. E. I. Boukili, D. Patigny, S. Dridi, D. Cartier and J. Lévy, “The rearrangement of some cyclopentanone-aryloximes: synthesis of (±) -aplysin, (±) -filiformin and of their debromo analogues,” *Tetrahedron*, vol. 47, no. 48, pp. 10003-10014, 1991.

[2] D. C. Harrowven, M. C. Lucas and P. D. Howes, “The synthesis of a natural product family: from debromoisolaurinterol to the aplysins,” *Tetrahedron*, vol. 57, no. 2001, pp. 791-804, 2001.
